# Predicting the protein half-life in tissue from its cellular properties

**DOI:** 10.1371/journal.pone.0180428

**Published:** 2017-07-18

**Authors:** Mahbubur Rahman, Rovshan G. Sadygov

**Affiliations:** Department of Biochemistry and Molecular Biology, Sealy Center for Molecular Medicine, The University of Texas Medical Branch, Galveston, Texas, United States of America; University of Edinburgh, UNITED KINGDOM

## Abstract

Protein half-life is an important feature of protein homeostasis (proteostasis). The increasing number of *in vivo* and *in vitro* studies using high throughput proteomics provide estimates of the protein half-lives in tissues and cells. However, protein half-lives in cells and tissues are different. Due to the resource requirements for researching tissues, more data is available from cellular studies than tissues. We have designed a multivariate linear model for predicting protein half-life in tissue from its cellular properties. Inputs to the model are cellular half-life, abundance, intrinsically disordered sequences, and transcriptional and translational rates. Before the modeling, we determined substructures in the data using the relative distance from the regression line of the protein half-lives in tissues and cells, identifying three separate clusters. The model was trained on and applied to predict protein half-lives from murine liver, brain and heart tissues. In each tissue type we observed similar prediction patterns of protein half-lives. We found that the model provides the best results when there is a strong correlation between tissue and cell culture protein half-lives. Additionally, we clustered the protein half-lives to determine variations in correlation coefficients between the protein half-lives in the tissue versus in cell culture. The clusters identify strongly and weakly correlated protein half-lives, further improves the overall prediction and identifies sub groupings which exhibit specific characteristics. The model described herein, is generalizable to other data sets and has been implemented in a freely available R code.

## Introduction

**Fig 1:**
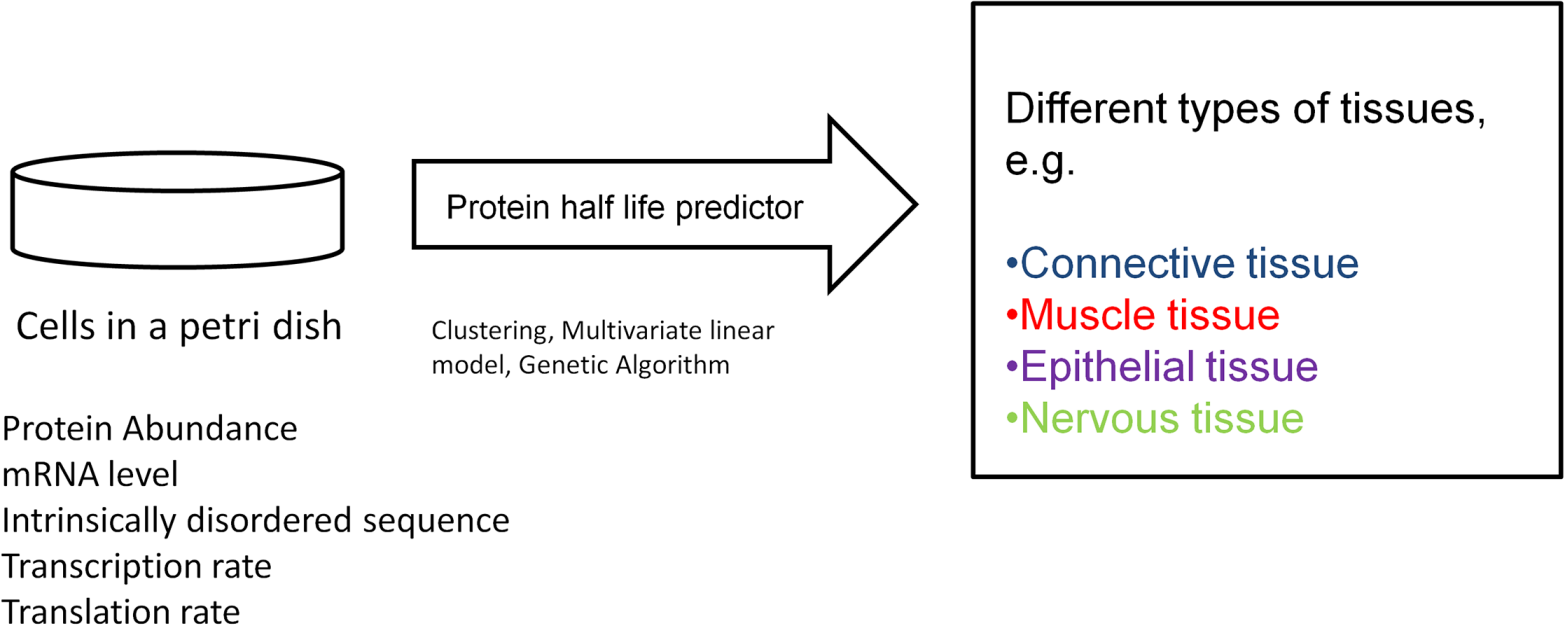
A schematic diagram of *in vivo* protein half-life prediction from cellular properties.

Proteostasis is a cellular process that includes control of concentrations, conformations, binding interactions, and locations of individual proteins[1]. Proteostasis integrates into other cellular processes such as (external or internal) signal response, cellular proliferation, and aging. It enables cells to change their physiology for successful organismal development and aging while under constant challenges from intrinsic and environmental factors. An important characteristic of proteostasis is the turnover rate of a protein (half-life). New technological advances in proteomics field are enabling researchers to profile the proteome dynamics of cell lines[2, 3], tissues, and living organisms in high throughput experiments[4], allowing for half-life estimations for a large number of proteins[5]. These experiments create new opportunities for inferring the networks and pathways controlling cellular proteostasis and assist with understanding the sequence of regulatory events that lead to the integration of cellular processes including gene expression, translation, post-translational protein modifications, and sub cellular localization. However, the analysis of the time course data from metabolic labeling experiments, especially generalization of the results from cell lines to the tissues which is required for such studies, poses several new challenges in bioinformatics, statistical data processing, and modeling. While proteome dynamics data from cell lines is becoming readily available, the labeling of living organisms is expensive and laborious. In addition, half-life measurements *in vivo* are meaningful only for relatively long living proteins as it takes a few hours for the administered labeling to be incorporated into a tissue in the body. However, this limitation is not present in cultured cells, allowing half-lives as short as one to two hours to potentially be measured. Therefore, computational techniques are needed to map the observations from cell lines to the corresponding tissues. Another challenge, though not addressed here, is that tissues are composed of different cell types, therefore requiring the combination of protein information from multiple cell types. In this study, we make a first attempt at predicting protein turnover rates in tissues from their cellular properties (e.g. Fig 1), and propose a multivariate linear model[6].

The model employs several cellular properties of proteins (e.g. protein half-life in the cell, abundance, length, mRNA level, transcriptional and translational rates, and segments of intrinsically disordered sequences) as explanatory variables, and the protein half-life in tissue as the response variable. The model is trained on randomly selected data sets by minimizing an objective function that is associated with the predictive results[7–9]. We have applied the genetic algorithm[10] (GA) to minimize the objective function. The GA returns the optimized values of model parameters which are then used to predict half-lives for the rest of the proteins.

To reduce the deviation between the protein half-lives in tissues and cells, we first determine substructures (clusters) in the protein half-lives (cellular and tissue) data. The clustering is based on the relative distance of a protein half-life from the linear regression line[11] between the protein half-lives in tissues and cells. Each cluster has its own multivariate linear model and associated parameters [6].

## Data

We used publicly available *in vivo* data sets from murine liver[7], brain[7], and heart[12], and *in vitro* data sets from murine fibroblast (NIH3T3[2]), and myoblast (C2C12[13]) cell lines for protein half-lives. The *in vivo* experiment used Nitrogen-15 (^15^N) isotope labeling in the murine brain and liver study[7] and heavy water labeling in the heart[12] study. The cell lines studies used stable isotope labeling with amino acids in cell cultures (SILAC)[14]. In these data sets, there were 434 liver proteins common between cell culture and liver tissue and 354 brain proteins common between cell culture and brain tissue. Of these, 366 common liver proteins and 346 common brain proteins have longer half-lives in the tissue than in the cell cultures (e.g. NIH3T3[2]). We have used these common protein data sets to train and validate the multivariate linear model of the protein half-life prediction. Finally, we have applied the model to other two data sets; one from the *in vitro*[8] C2C12[13] myoblasts and another from the *in vivo* murine heart experiment[12] (Supporting information).

## Methods

**Fig 2:**
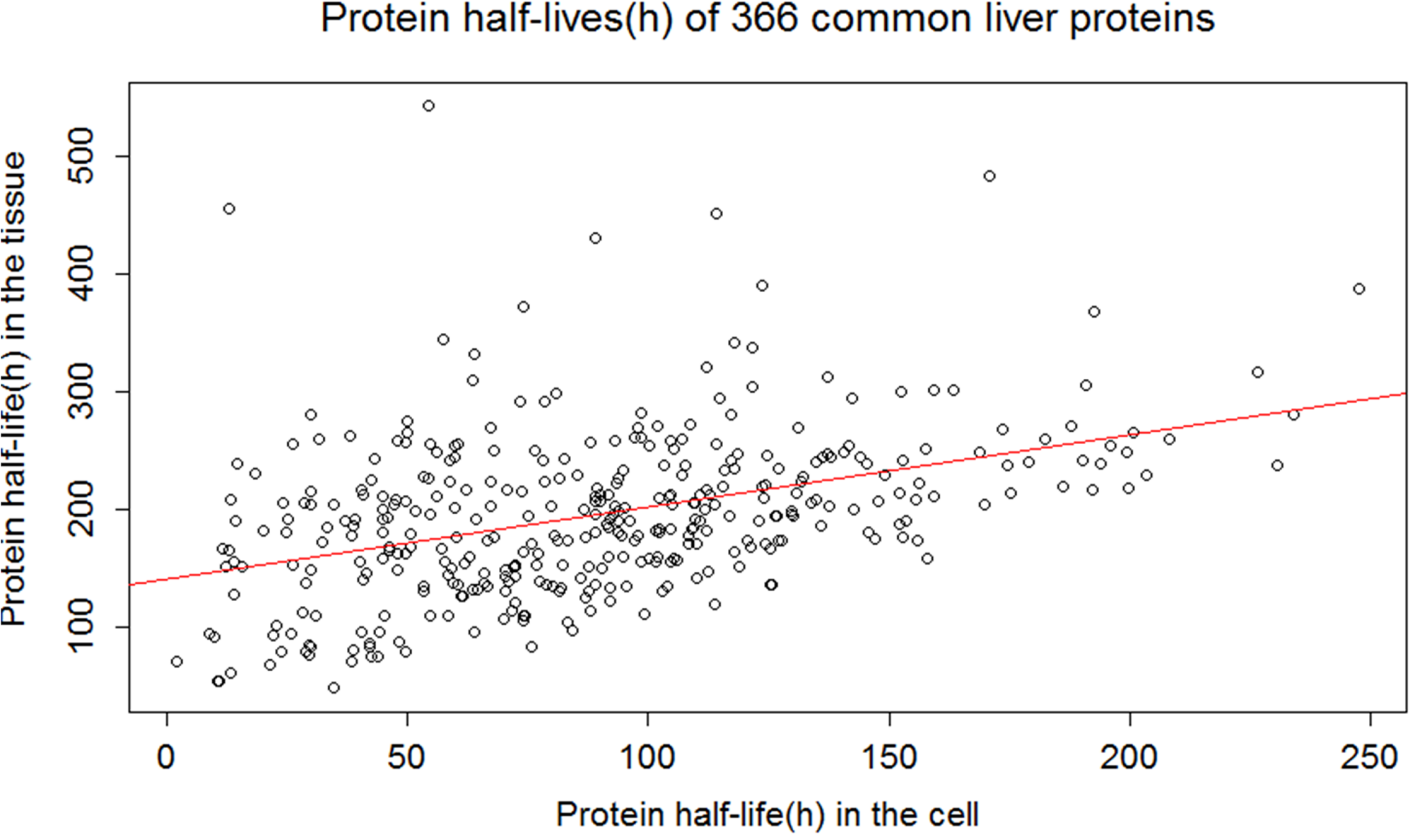
Linear regression between the half-lives of proteins present in the murine liver tissue and cell culture (e.g. NIH3T3[2]) data sets.

We developed a model using proteins which have longer half-lives in the tissue than in cell culture and are common in both (91% of all data). However, the model is also applicable to those common proteins that have shorter half-lives in the tissue than in cell culture (see Results). For a first attempt, we were interested in predicting the protein half-lives for the former group (longer tissue half-lives). Hence, we created our protein data sets from the experimental data sets[7–9] following the first assumption. We applied a linear regression[11] model to the common protein half-life data sets[2, 7] (Fig 2 and S1 Fig) to first understand their linear relationship.

The linear regression model has the following form:

$$Y_{t}=mX_{t}+w_{t}$$ (1)

Here, *Y*_*t*_ is the protein half-life in the tissue, *X*_*t*_ is protein half-life in the cell culture, *m* is the slope, and *w*_*t*_ is the intercept of the linear model.

The half-lives of proteins are scattered around the linear regression line (Fig 2 and S1 Fig), demonstrating that a single, multivariate linear model cannot be a good predictor of protein half-life. To improve the model prediction, we needed a method to systematically cluster the data sets. Therefore, we searched for substructures in the protein half-life distribution as a way to cluster the data sets. The answer was found in the correlation coefficient between the protein half-lives at the tissue and cellular levels.

**Fig 3:**
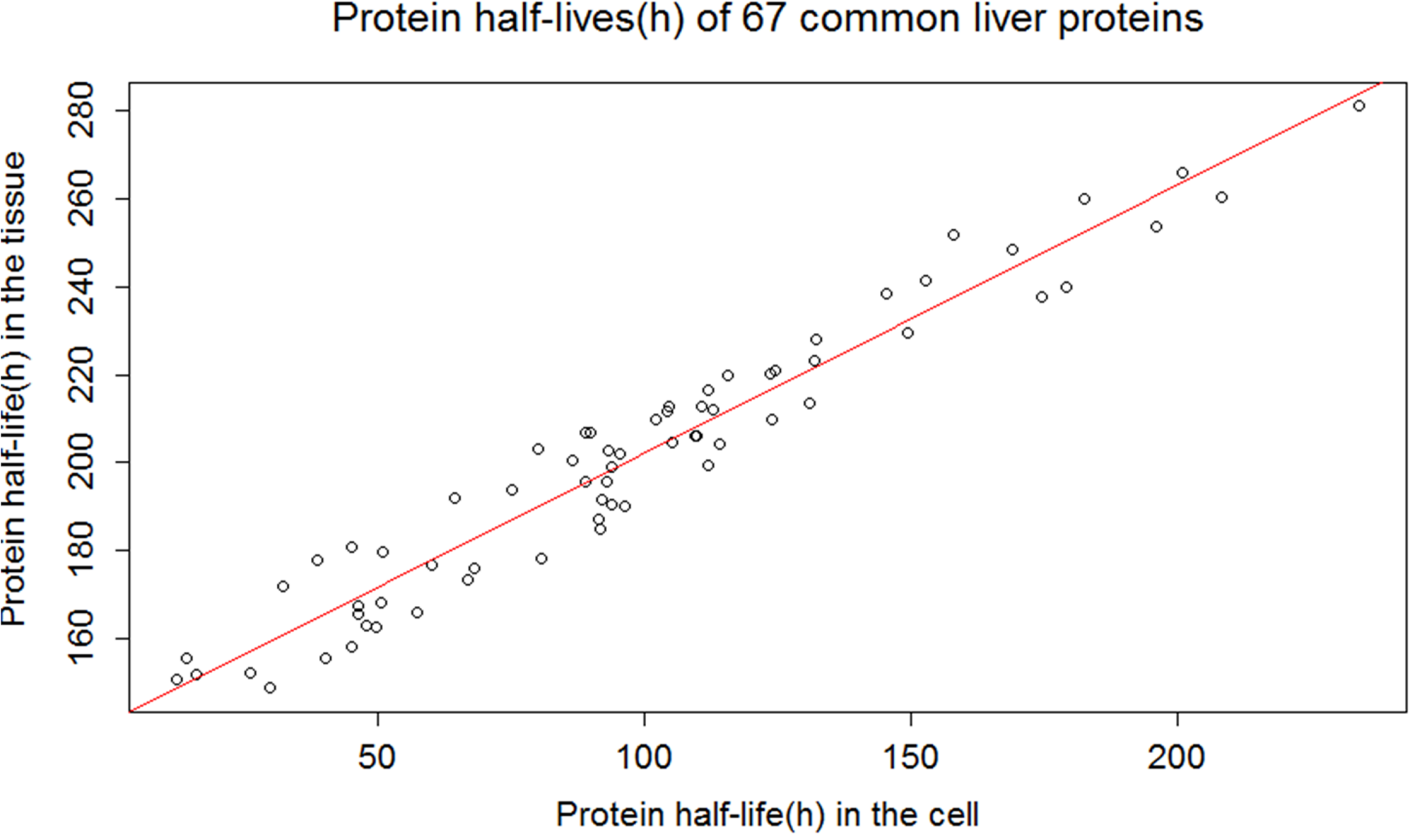
Common liver protein subset with the highest correlation coefficient between the protein half-life in the tissue and cells.

We clustered the proteins based on strongly and weakly correlated protein half-lives in cells and tissues. The approach provided a systematic analytical perspective for clustering, while improving the prediction of protein half-lives in tissues. We found that the correlation coefficient is the highest for some common liver proteins, which are the nearest to the regression line (red line in Fig 2). Within 10% deviation from the regression line, we found 67 common liver proteins that have correlation coefficients of 0.97 (Fig 3). This observation is consistent for the brain and heart proteins (S2 and S3 Figs) as well.

**Fig 4:**
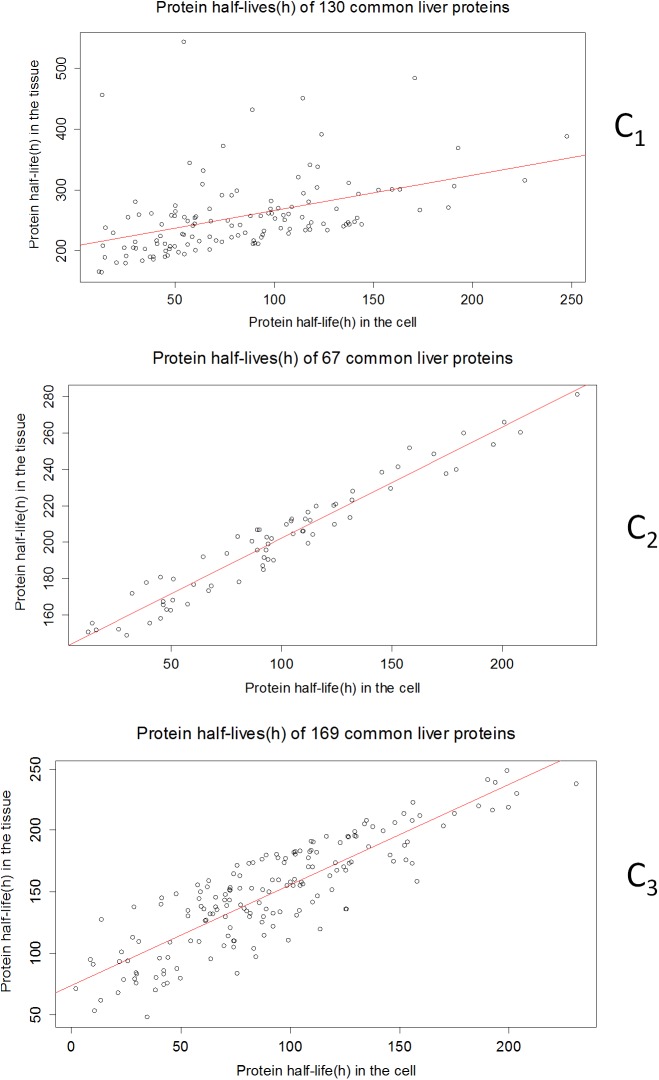
The clustering scheme of common liver protein data sets. The protein clusters form from the linear regression line (Fig 2). C_1_ has very long-living half-life proteins while others (C_2_, C_3_) have short-living proteins.

The cluster within 10% deviation from the regression line consists of those 67 proteins (Fig 3); additionally, there are two other protein clusters, one which exists above and one below the 10% deviation from the regression line cluster (Fig 4). This result leads us to the protein half-life clustering for strongly correlated (e.g. first cluster) and weakly correlated (e.g. later two clusters) half-lives. If we group the data set ((*X*_*c*_, *Y*_*c*_), *c* ∈ {*C*_1_,*C*_2_,*C*_3_}) into three clusters (e.g. {*C*_1_,*C*_2_,*C*_3_}), then one cluster is above 10% deviation (C_1_ in Fig 4), one is between 10% deviation (C_2_ in Fig 4), and the last one is below 10% deviation from the regression line (C_3_ in Fig 4). This clustering scheme has been also applied to the other two data sets (common brain and heart protein data sets) (S2 and S3 Figs respectively).

There are 130 proteins in C_1_, 67 proteins in C_2,_ and 169 proteins in C_3_ (N_c_ used below). Each data set has the protein half-lives at tissue and cellular levels along with several other protein properties. We built a multivariate linear model[6] between the protein half-life in the tissue and cells for each of the clusters using cellular protein properties. We have assumed that the protein half-life in the tissue can be predicted through the linear combination of the protein half-life in the cell and other protein properties:

$$Y_{c}^{s}=w_{Cell\_half-life}X_{c}^{s}+w_{P\_length}{PL}_{c}^{s}+w_{P\_abundance}{PA}_{c}^{s}+w_{I\_sequence}{ID}_{c}^{s}+w_{mRNA}{MR}_{c}^{s}+w_{Transcription}{TR}_{c}^{s}+w_{Translation}{TL}_{c}^{s}+w_{c}$$ (2)

where the weight vectors (e.g. w_Cell_half-life_, w_P_length_, w_P_abundance_, w_I_sequence_, w_mRNA,_ w_Transcription,_ w_Translation_) ϵ (0,1), w_c_ is the intercept from the linear regression (Eq 1 and Table 1) and s contains randomly selected one-third of total data sets (e.g. (X^s^_c_, Y^s^_c_)) of each cluster. Protein properties such as abundance (PA), mRNA level (MR), transcriptional (TR) and translational rates (TL) from the cell line study[2], and intrinsically disordered sequences (ID) for each protein have been calculated using the IUPRED software[15]. Protein lengths were calculated from the UniProt database. All protein properties were transformed to log_e_ for consistency in the calculations (e.g. a precision of 4 digits after the decimal). Each cluster (e.g. *c* ∈ {*C*_1_,*C*_2_,*C*_3_}) has its own protein half-life predictor (Eq 2).

**Table 1:** Coefficients of the linear regression between the common liver protein half-life in the tissue and cell of each cluster (Fig 4).

Cluster	w_c =_ Intercept (h)	Regression coefficient	P-value
C_1_	208.3428	0.5808	2.39e-07
C_2_	141.28857	0.60863	2.2e-16
C_3_	73.38162	0.81970	2.2e-16

Through predictive iterations, the model ascertains protein half-life from cell culture data using randomly selected proteins, and then predicts the protein half-life in the tissue using a learning scheme for the rest of the proteins in each cluster. In the Results section, we have provided a comparative analysis of the protein half-life prediction for each of the clusters. To compute the distribution of the weight vectors through a learning scheme and to understand their effect on the protein half-life prediction, we used a learning algorithm. The following objective function was used:

$$E_{min}(w_{Cell\_half-life},\;w_{P\_length},\;w_{P\_abundance},w_{I\_sequence},w_{mRNA},\;w_{Transcscription},w_{translation})={}_{\;s=1}^{1/3Nc}\sum \left(abs(Y_{c}^{s}-Y_{c})/Y_{c}\right)$$ (3)

We have applied the genetic algorithm (GA)[10] to minimize Eq 3. The GA has been implemented with a freely available genetic algorithm package[16] in the R environment, using the default settings (e.g. mutation rate, crossover rate etc.). The GA provides the distribution of the weight vectors (e.g. w^G^_Cell_half-life_ w^G^_P_length_, w^G^_P_abundance_, w^G^_I_sequence_, w^G^_mRNA_, w^G^_Transcription_, w^G^_Translation_ in S2 and S3 Tables). Next, the distribution of the weight vectors is used to predict the protein half-life in the tissue level for the rest (two-third of total data sets) of the proteins (e.g. ($X_{c}^{/},Y_{c}^{/}$)) in each of the clusters.

As a measure of performance of the model, we used the following percentage of error (PE) equation to measure the relative deviation of protein half-life prediction in tissue from the corresponding experimental value:

$$PE=|Y_{c}^{v}-Y_{c}^{/}|/Y_{c}^{/};\;Y_{c}^{v}=w_{Cell\_half-life}^{G}X_{c}^{/}+w_{P\_length}^{G}{PL}_{c}^{/}+w_{P\_abundance}^{G}{PA}_{c}^{/}+w_{I\_sequence}^{G}{ID}_{c}^{/}+w_{mRNA}^{G}{MR}_{c}^{/}+w_{Transcription}^{G}{TR}_{c}^{/}+w_{Translation}^{G}{TL}_{c}^{/}+w_{c}$$ (4)

The use of PE (e.g. PE% in Table 2) is to better understand the performance of the model that includes the deviation (e.g. 5%, 10%, 20% and 30%) of the predicted half-life in the tissue from the experimental observation.

**Table 2:** Performance analysis of the protein half-life prediction of the model with two types of protein properties (e.g. PCH, ACH) using two-thirds (e.g. ($X_{c}^{/},Y_{c}^{/}$)) of total data sets (common liver proteins) of each cluster. All protein properties are designated by ACH, and positively-correlated proteins are designated by PCH. The best result provided is the **C**_**2**_ cluster. It has predicted 44% of protein half-lives between 10% deviation from the experimental value.

Cluster	PCH					ACH							
	Deviation	5%	10%	20%	30%	Deviation	5%		10%		20%		30%
C_1_	PE%	19%	36%	66%	83%	PE%	21%		32%		65%		83%
**C**_**2**_	**PE%**	**24%**	**44%**	**62%**	**77%**	**PE%**	**22%**		**44%**		**62%**		**77%**
C_3_	PE%	8%	15%	33%	45%	PE%	8%		15%		35%		48%

The values of the correlation coefficients show that some of the protein properties are correlated either positively or negatively (S1 Table) with the protein half-life in the tissue. This has an impact on the prediction outcome which is discussed in the Results section. Hence, we analyzed the performance of the model with (a) ACH: all protein properties and (b) PCH: positively-correlated protein properties along with their optimized weight vectors obtained from the GA optimization (S2 and S3 Tables).

## Results

We are presenting the analysis of the performance of the model, the effect of clustering on the protein half-life prediction, and half-life prediction of uncommon proteins. The analysis focuses on the correlation coefficients between protein half-lives in the tissue and cell cultures, as the half-life clusters are formed based on the correlation coefficients. This also leads to the identification of common protein half-life characteristics of each cluster (S4 Table). We have observed that these characteristics are common to the murine proteins from liver, brain, and heart. Additionally, we have analyzed biological/biochemical properties of proteins from the protein database[17].

### Model performance for each cluster

In the liver data set, each cluster varies in the number of proteins and types of either positively or negatively-correlated protein half-life properties (S1 Table). C_1_ has the largest intercept (Table 1) of the linear regression and highest number of positively-correlated protein properties (S1 Table). This observation is true for the brain and heart data sets (S5 and S10 Tables, respectively). On the other hand, C_3_ has the smallest intercept (Table 1) and least number of positively-correlated protein properties (S1, S5, S6, S10 and S11 Tables).

Based on the PE, the best result of the protein half-life prediction is observed for C_2_ (44% of prediction is within 10% deviation from the experimental value in Table 2). This has been consistent for the other two tissue types’ (brain and liver) data sets (S9 and S14 Tables) as well. C_2_ has the largest proportion of protein half-lives close to the regression line (Fig 4) and these protein half-lives have the strongest correlation coefficients (0.97) between the tissue and cell culture data. The model parameters (weight vectors etc.) associated with C_2_ can be used to predict proteins with strongly correlated half-lives in the tissue from cell culture experimental data.

On the other hand, C_1_ and C_3_ (of the liver data set) have protein half-lives that deviate from the regression line (e.g. Fig 4). For these clusters, the model does not perform as well as it does for C_2_. This has been also consistent for other two data sets (S9 and S14 Tables). The predictions can be improved if one does additional clustering above and below 10% deviation from the regression line for each of the two clusters (clustering inside the cluster[18]). This clustering inside the clusters generates clusters that have less deviations between the regression lines and the protein half-lives (e.g. C_2_ of liver, brain and heart). Additionally, clustering improves the overall prediction (e.g. prediction improves more than three times, two times and ten times for common liver, brain, and heart protein respectively). It also provides an opportunity for protein half-life prediction for strongly correlated (C_2_) and weakly correlated proteins (C_1_ and C_3_) in the tissue and from the cell.

The cell culture protein half-life has the highest positive correlation with the tissue half-life than the rest of the protein properties of each cluster (S1 Table). Some protein properties have a negative correlation coefficient with the protein half-life in the tissue. However, these are very weak (Cor(Tissue half-life, mRNA level) in C_2_, C_3_ of S1 Table). We have omitted these variables (reduced model) while predicting half-lives (PCH column in Table 2) to compare with the result using all protein properties in the full model (ACH column in Table 2).

The reduced model predicts consistently with the full model (Table 2 and S9 and S14 Tables), possibly showing that the prediction may require fewer protein properties. We found that C_3_ has shorter protein half-lives and the smallest number of positively-correlated protein properties (C_3_ row in S1 Table). Alternatively, C_1_ has longer protein half-lives and the highest number of positively-correlated protein properties. For both C_1_ and C_3_ the reduced model is consistent with the full model for protein half-life prediction. However, for the shorter half-life C_3_ cluster, the percentage predicted between 10% deviation from the experimental value is around half that of C_1_ (15% and 32% respectively).This seems to indicate that the multivariate linear model needs larger number of protein properties for predicting weakly correlated protein half-lives. This observation is also true for the brain and heart data sets (C_1_ rows in S9 and S14 Tables, respectively).

### Comparison with other studies

Our results are consistent with previous research findings regarding half-lives of short-lived proteins. Corroborating the previous work, an important feature of C_3_ is the negative correlation between the number of disordered sequences and the protein half-life in the tissue (S1 and S11 Tables) and these proteins are short-living (Fig 4 and S3 Fig). On the other hand, the brain proteins that belong to C_3_ have a larger intercept and longer half-lives (S5 and S6 Tables). Hence, these proteins provide positive correlation between the number of disordered sequences and the protein half-life in the tissue as it is observed in C_1_ of common liver proteins (S1 Table). We have observed similar characteristics of the correlation coefficients between the number of ubiquitination sites[19] and the protein half-life (S15 Table). The short-living proteins (C_3_ of common liver and heart proteins) have more ubiquitination sites (S15 Table) and long-living proteins have fewer ubiquitination sites. More ubiquitination sites are expected to lead to faster degradation of proteins via the ubiquitin-proteasome proteolytic pathway. Our model can identify these proteins in the cell line data sets and predict their half-life in the tissue.

We have applied our model to both *in vivo* and *in vitro* murine proteins. However, a previous study[20] found common properties between the number of intrinsically disordered segments and the protein half-life in yeast, *in vitro* human proteins, and *in vitro* mouse proteins which we have used. Hence, we believe that this multivariate linear model can be potentially applicable to yeast and human proteins as well.

### The genetic algorithm retrieves the correlation coefficients

We have used the GA to minimize the objective function (Eq 3). The value of the weight vector (w^G^_Cell_half-life_) that is associated with the protein half-life in the cell in Eq 2, combined with the weight vector received from the GA (second column in S2 and S3 Tables) are close to the regression coefficient (third column in Table 1) of each cluster. This has been a common feature of the GA when there is less deviation (e.g. 10%) between the protein half-life and the regression line (e.g. clusters: C_2_ and C_3_ in S2 Table, and C_2_ in S12 and S13 Tables).

Additionally, the GA uncovers the importance of other protein properties. For example, the GA provides larger weight on transcription rate (w^G^_Transcription_ of C_3_ in S2 Table) for common liver proteins, and protein abundance and mRNA level (w^G^_mRNA_ and w^G^_P_abundance_ of C_2_ in S13 Table) for common heart proteins. However, the protein half-life is scattered around the regression line of C_2_ for common brain proteins (S2 Fig). Hence, GA does not provide any significant weight vector for the protein properties of these common brain proteins except for the protein half-life in the cell (S5 Table). Because all of the aforementioned protein properties belong to PCH (e.g. they have positive correlation with the tissue half-life), we conclude that the model indicates the importance of PCH for predicting strongly and weakly (though total number of PCH increases) correlated protein half-lives.

### Neural network (NN) to classify proteins into the clusters

Our modeling uses clustering of protein half-lives into three clusters: the first cluster has the proteins which have much longer half-lives in the tissue than in the cell; the second cluster has the proteins which have correlated half-lives at these two levels; finally, the third cluster has the half-lives of those proteins which have been influenced by the intrinsically disordered sequences directly[20]. Hence, each cluster has its own features, importance, and biological/biochemical perspective.

**Fig 5:**
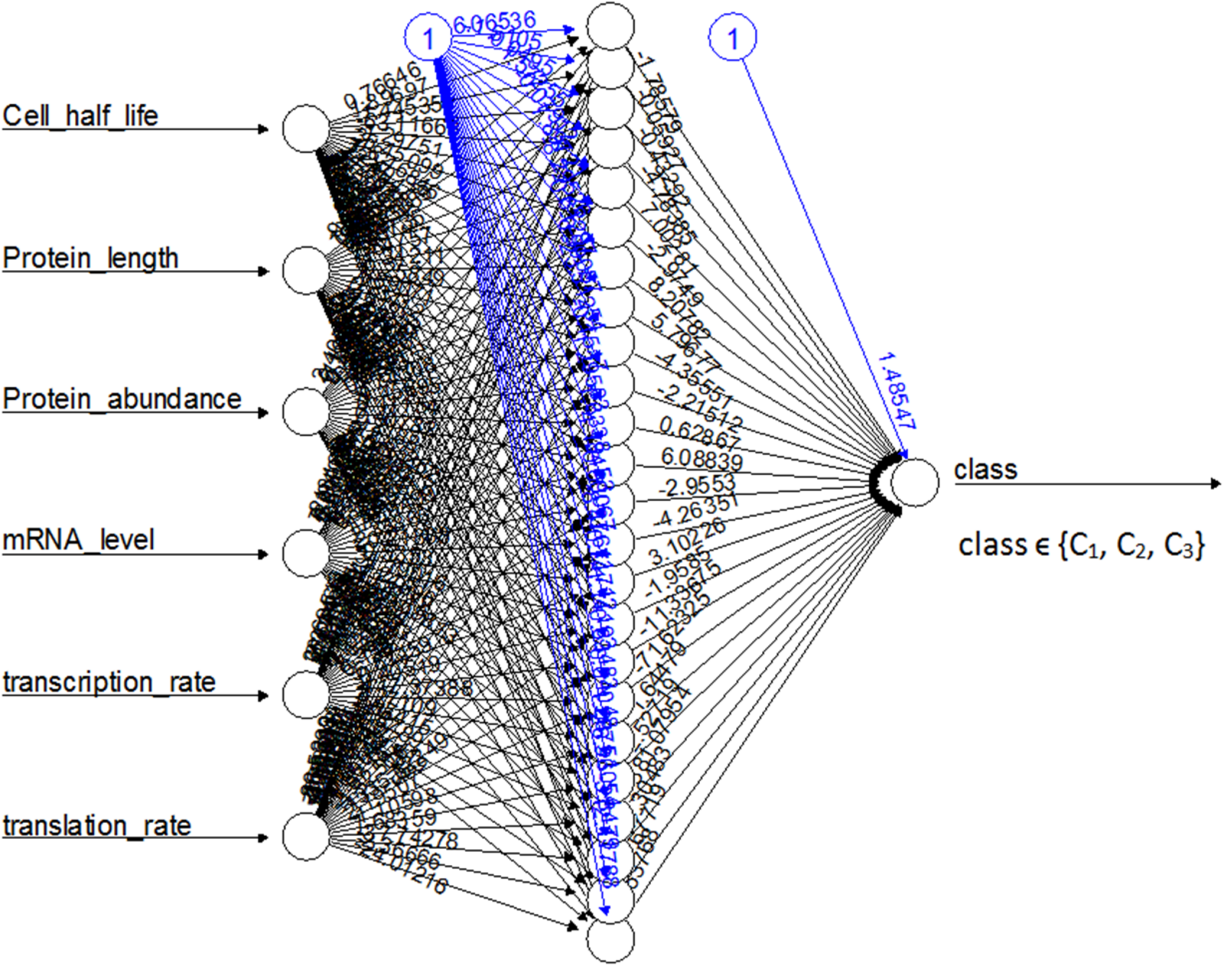
Artificial neural network between the protein properties and clusters.

We have designed a neural network[21] (Fig 5) between the cell-line data set[2] and three clusters of common liver proteins to train a neural network for classifying proteins into clusters based on their cellular properties. We have tested this network with a number of neurons and found an optimal set with 24 neurons between the input (protein properties) and output (clusters/classes) layers. This network was able to classify the highest number (78%) of common liver proteins successfully. Using this network, we can classify an unknown protein into one of the clusters, then predict its half-life with the model by using the optimized parameters for that cluster.

We calculated the probabilistic distribution of the clusters and used this while clustering the proteins with the neural network (NN). The black lines show the connections between each layer and the weights on each connection, while the blue lines show the bias term added in each step[21]. The bias can be thought as the intercept of a linear model. The NN provides the probabilistic distribution of the clusters which we have used to cluster the uncommon proteins.

### Predicting uncommon protein half-lives

**Fig 6:**
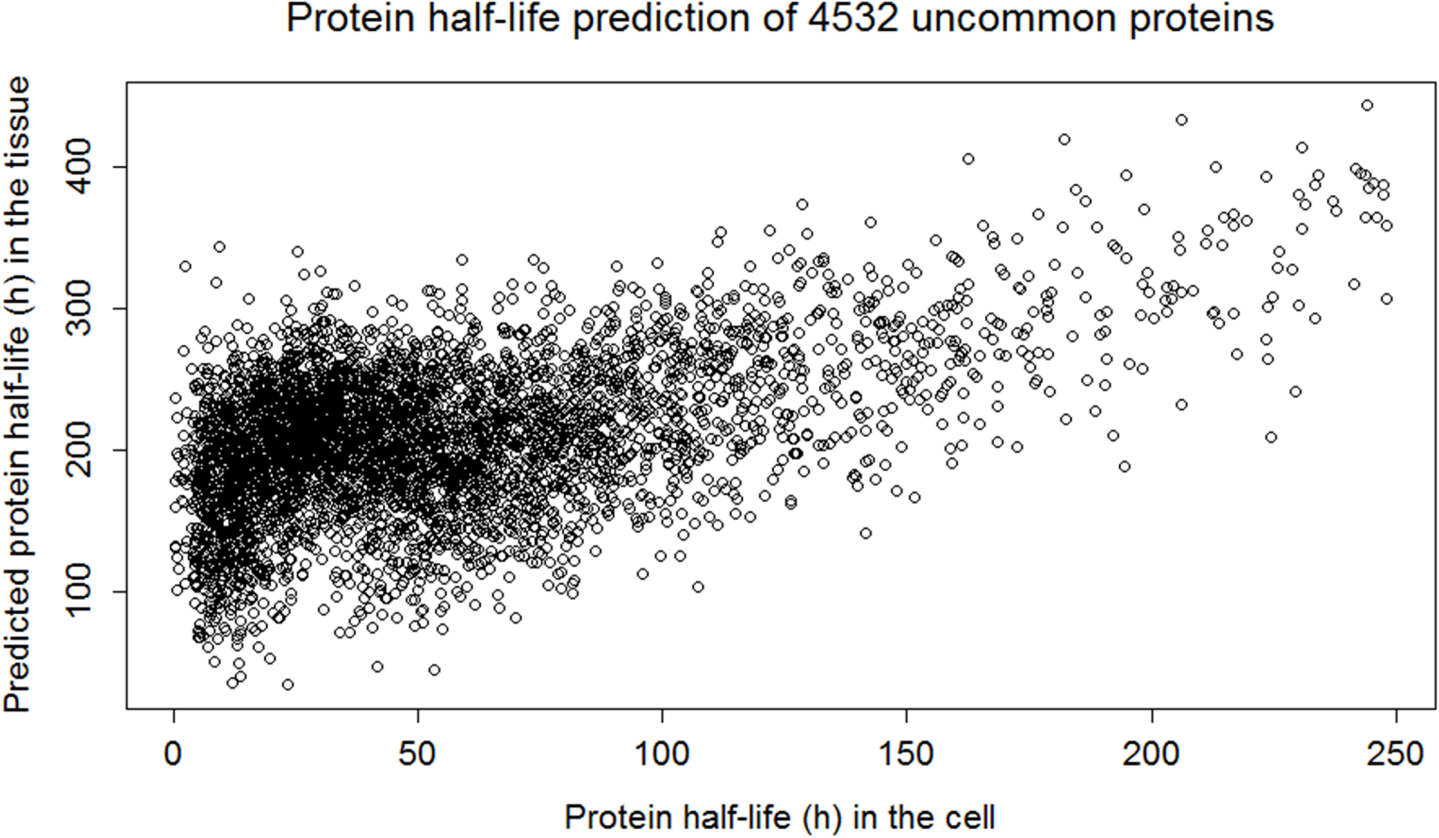
Uncommon protein half-life prediction with noise.

We used the multivariate linear model and optimized weight vectors for each of the clusters for predicting the protein half-life of uncommon proteins (S4 Fig). A cluster for a protein is determined from the NN. The predicted half-lives produce three lines since the prediction follows the multivariate linear model of the clusters. We added normally distributed noise to the predicted half-lives to account for the variability of protein half-lives in the tissue, Fig 6. This noise has been generated from the mean and standard deviation of common protein half-life in the tissue of the corresponding cluster.

#### Predicting shorter tissue half-lives than cell half-lives

We have proposed a model for the proteins which have longer half-lives in the tissue than in the cell-line. We found that 84% proteins from the liver and heart protein data sets and 97% proteins from the brain protein data sets have longer half-lives in the tissue than in cell culture. Since most of the available protein data sets have longer half-lives in the tissue than in the cell-lines, we selected those proteins in the linear model. The rest of the proteins which have shorter half-lives in the tissue than in the cell-line, can be explained with this model as well, if we disregard those proteins which have half-lives greater than 200 hours in the cell. And we can do this since the authors[2] of the cell-line study mention that very long (>200h) and very short (< 30 min) protein half-lives in the cell-line cannot be accurately quantified from the three time points (i.e. 1.5h, 4.5h, 13.5 h) which they used for metabolic labeling.

Once we remove proteins with half-lives greater than 200 h, only 6% of common liver proteins have longer half-lives in the cell-line. We classify 83% of these proteins by using the above described NN. These proteins also exhibit a linear relationship of half-lives (S5 Fig) from the cells and the tissue which we can fit with our model.

### Protein classification through database search

We looked at the proteins from each of our clusters in the PANTHER database[17] to identify biological and biochemical properties of the corresponding proteins (S6–S11 Figs). Most of the long-living proteins (C_1_) group as nucleic acid binding (tallest bar in S6 Fig). However, most of the short-living proteins (C_3_) belong in the oxidoreductase category (tallest bar in S10 Fig). These short-living proteins also belong to the ubiquitin-proteasome pathway (Longest bar in S11 Fig) which exhibit faster degradation[19].

## Conclusion

We have provided the first study of predicting the protein half-life at the tissue level from the cellular level. The model is simple, easy to implement and will be applicable to other tissues and cell line experimental data sets. We have analyzed the linear relationships between the protein half-life in the tissue and cell by using the multivariate linear model along with clustering. The clustering reveals linear and correlation coefficient based relationships between the protein half-lives and protein properties along with improvement of the prediction.

The ability to predict the protein half-life at the tissue level from the cellular perspective can help to understand the overall effect and interplay of protein properties and identify novel variables that play significant roles in proteostasis[22]. Proteostasis has been shown to be important in different diseases[23], for biomarker analysis, and drug design[24].

Other future aspects of this research include predicting the degradation pathway of the protein by ubiquitination[25] and determining the effects of transcriptional and translational synthesis rates for predicting protein half-life in tissues. As the amount of experimental data increases, more data will be available to validate the model’s prediction and uncover new relationships for predicting tissue biology from the cellular perspective.

## Supporting information

S1 Fig Linear regression between the half-lives of proteins present in the murine brain tissue and cell culture (NIH3T3) data sets. Shown are the data for half-lives of 346 common proteins. The red line is the line of linear regression between the protein half-lives in the tissue and cell lines.
(TIF)

S2 Fig The clustering scheme of common brain protein data sets. The protein clusters are obtained from the linear regression line (the red line in the left plot). The cluster C_1_ contains very long-living proteins while the other clusters (C_2_, C_3_) contain short-living proteins.
(TIF)

S3 Fig The clustering scheme of common heart protein data sets (779 proteins). The protein clusters are generated using the linear regression line (the red line in the left plot). C_1_ contains very long-living proteins while the others (C_2_, C_3_) contain short-living proteins. The clustering improves the correlations between half-lives of proteins in the tissue and cell lines.
(TIF)

S4 Fig Protein half-life prediction of 4532 uncommon proteins in the liver (hour). The half-lives of the uncommon proteins were predicted using a multivariate linear model and optimized weight vectors for each of the three clusters. For each protein its cluster was determined using the NN.
(EMF)

S5 Fig 26 Common liver protein half-lives (hour). Scatter plot of common liver proteins that have longer half-lives in the cell-line data (shown are proteins with half-lives less than 200 hours). These proteins exhibited a linear relationship of half-lives which we could fit with our model.
(EMF)

S6 Fig Protein class analysis of the C_1_ cluster proteins using PANTHER database. Most of the long-living proteins belong to the class of nucleic acid binding proteins.
(TIF)

S7 Fig Pathway analysis of the C_1_ cluster proteins using PANTHER database. The most enriched pathway among the C_1_ cluster proteins was the integrin signaling pathway.
(TIF)

S8 Fig Protein class analysis of the C_2_ cluster proteins using PANTHER database. Most of the proteins from this cluster belong to the class of nucleic acid binding proteins.
(TIF)

S9 Fig Pathway analysis of the C_2_ cluster proteins using PANTHER database. For the proteins of the cluster C_2_ two pathways were enriched: 1) inflammation mediated by the chemokines and cytokines signaling pathway, and 2) the integrin signaling pathway.
(TIF)

S10 Fig Protein class analysis of the C_3_ cluster proteins (short half-life proteins) using PANTHER database. The most enriched for the proteins of this cluster was the oxidoreductase category. Also enriched were the nucleic acid binding and the enzyme modulator proteins.
(TIF)

S11 Fig Pathway analysis of the C_3_ cluster proteins (short half-life proteins) using PANTHER database. The short-living proteins belonged to the ubiquitin-proteasome pathway.
(TIF)

S1 Table Correlation coefficients among the common liver protein half-life in the tissue and all other protein properties for every cluster (Fig 4). (DOCX)

S2 Table Optimized weight vectors of all protein properties received from the GA Eq 3. (DOCX)

S3 Table Optimized weight vectors of positively-correlated protein properties received from the GA, Eq 3. Negatively-correlated protein properties (S1 Table) have 0 weights (e.g. w^G^_mRNA_ in C_1_,C_2_,C_3_.).
(DOCX)

S4 Table Common characteristics of common liver protein half-lives observed in three clusters (e.g. Fig 4). (DOCX)

S5 Table Coefficients of the linear regression between the common brain protein half-life in the tissue and cell of the clusters (S2 Fig). (DOCX)

S6 Table Correlation coefficients among the protein half-life in the tissue and protein half-life properties in the clusters (S2 Fig). (DOCX)

S7 Table Optimized weight vectors of all protein half-life properties received from the GA (3). (DOCX)

S8 Table Optimized weight vectors of positively-correlated protein half-life properties received from the GA (3). Negatively-correlated protein half-life properties (e.g. S6 Table) have 0 weights.
(DOCX)

S9 TablePerformance analysis of the protein half-life prediction of the model with two types of protein properties (ACH, PCH) using two-third (i.e. ($X_{c}^{/},Y_{c}^{/}$)) of total data sets (common brain proteins) of each cluster.**C_2_** provides the best result. It has predicted 76% of protein half-lives within 10% deviation from the experimental value.(DOCX)

S10 Table Coefficients of the linear regression between the common heart protein half-life in the tissue and cell of each cluster (S3 Fig). (DOCX)

S11 Table Correlation coefficients among the common heart protein half-life in the tissue and protein half-life properties in the clusters (S3 Fig). (DOCX)

S12 Table Optimized weight vectors of all protein half-life properties received from the GA (3). (DOCX)

S13 Table Optimized weight vectors of positively-correlated protein half-life properties received from the GA (3). Negatively-correlated protein half-life properties (e.g. S11 Table) have 0 weights.
(DOCX)

S14 TablePerformance analysis of the protein half-life prediction of the model with two types of protein properties (ACH, PCH) using two-third (i.e. ($X_{c}^{/},Y_{c}^{/}$)) of total data sets (common heart proteins) of each cluster.**C_2_** provides the best result. It has predicted 33% of protein half-lives within 10% deviation from the experimental value.(DOCX)

S15 Table Correlation coefficients between the protein half-life and ubiquitination. (DOCX)
