# Supplementary figures and images for: Predicting the protein half-life in tissue from its cellular properties
Source: PLoS One. 2017 Jul 18;12(7):e0180428. doi: 10.1371/journal.pone.0180428 (PMC5515413; doi:10.1371/journal.pone.0180428)

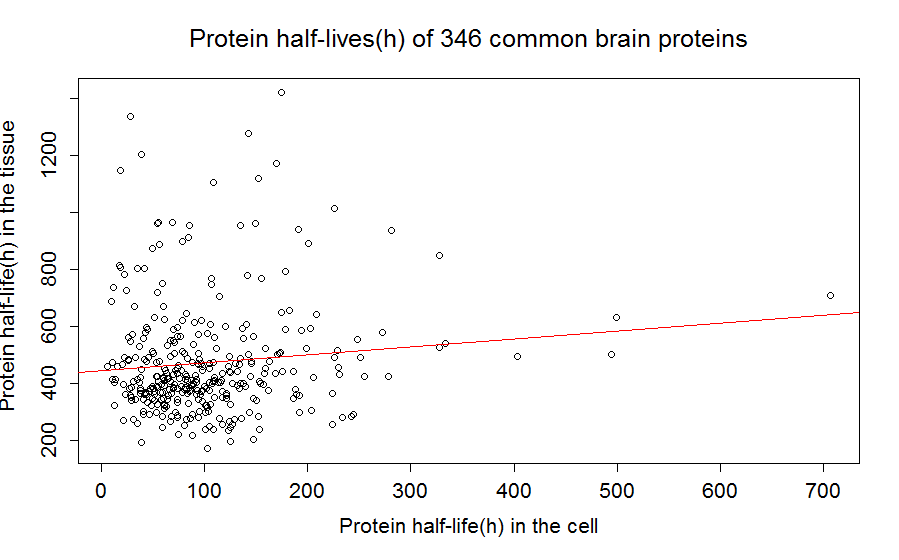

Supplement: S1 Fig — Shown are the data for half-lives of 346 common proteins. The red line is the line of linear regression between the protein half-lives in the tissue and cell lines. (TIF) [file pone.0180428.s001.tif]

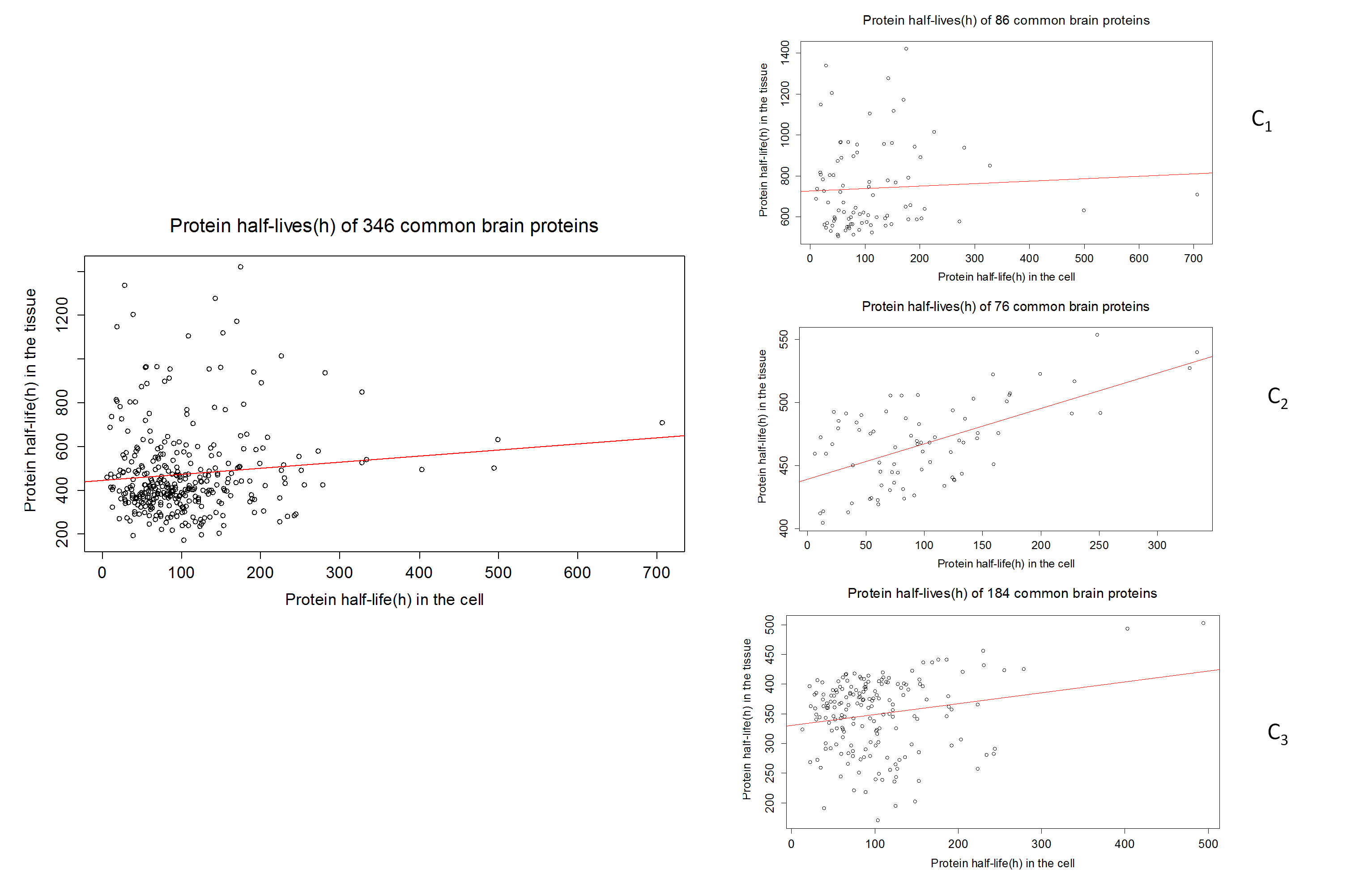

Supplement: S2 Fig — The protein clusters are obtained from the linear regression line (the red line in the left plot). The cluster C1 contains very long-living proteins while the other clusters (C2, C3) contain short-living proteins. (TIF) [file pone.0180428.s002.tif]

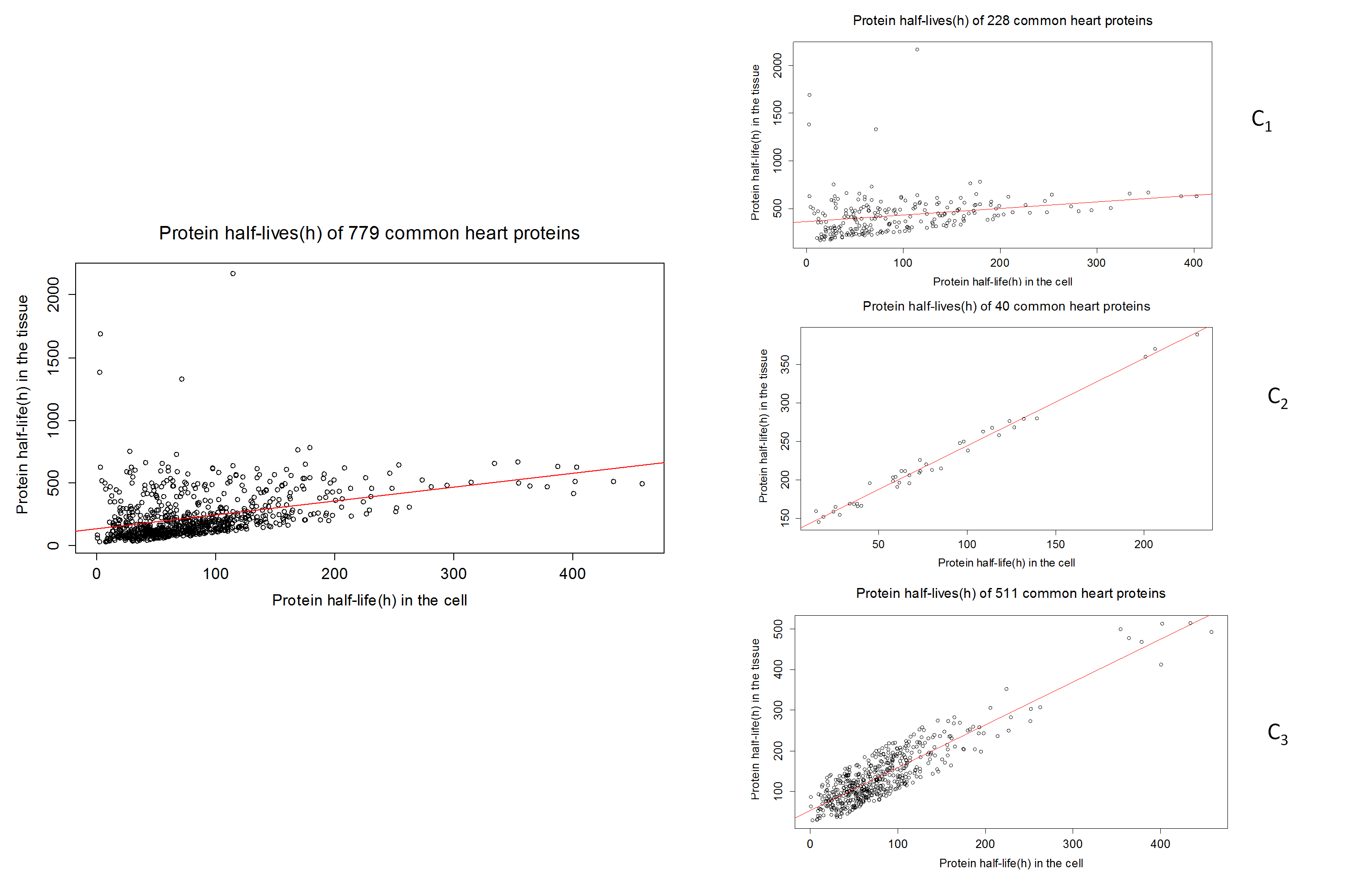

Supplement: S3 Fig — The protein clusters are generated using the linear regression line (the red line in the left plot). C1 contains very long-living proteins while the others (C2, C3) contain short-living proteins. The clustering improves the correlations between half-lives of proteins in the tissue and cell lines. (TIF) [file pone.0180428.s003.tif]

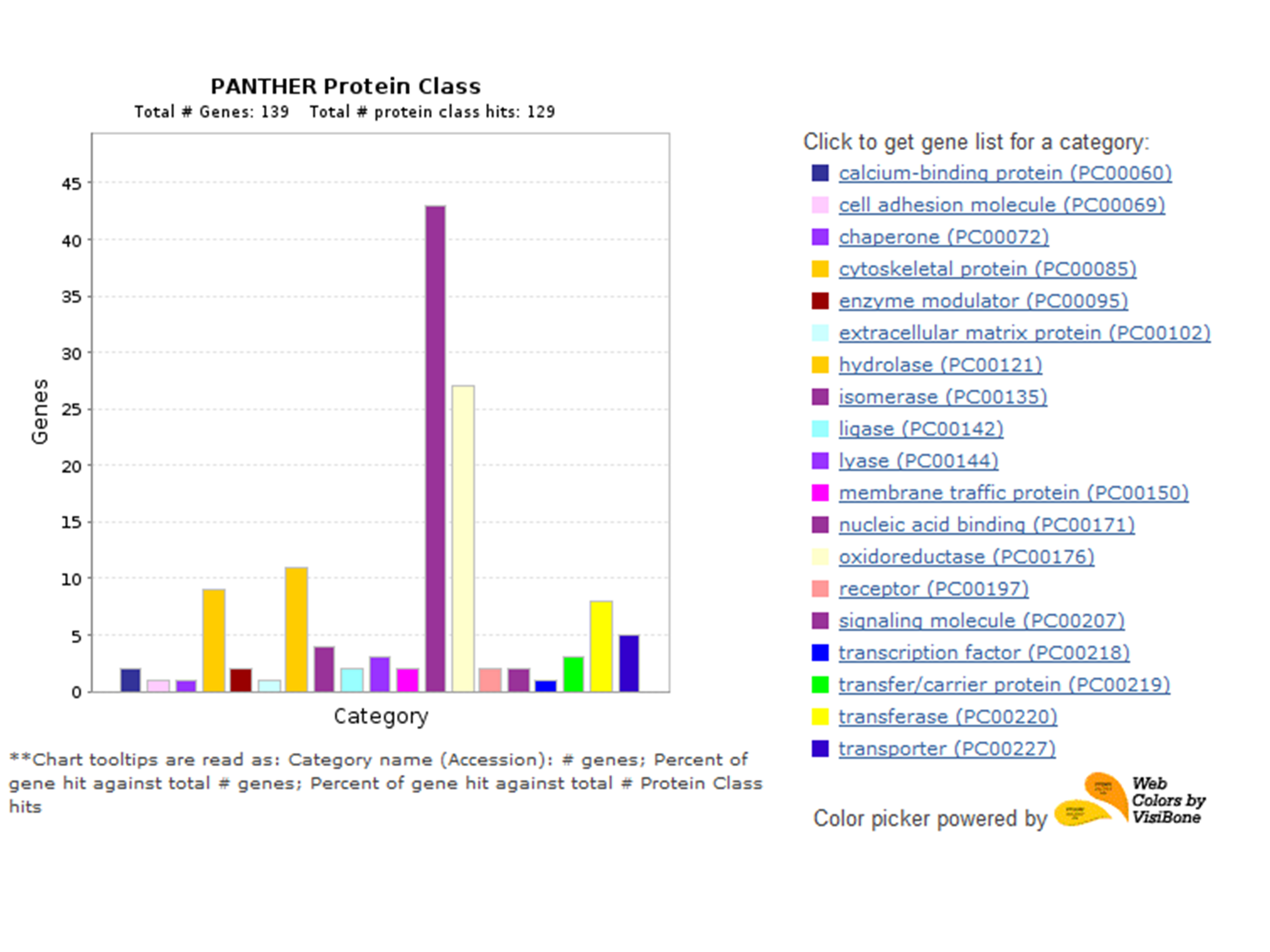

Supplement: S6 Fig — Most of the long-living proteins belong to the class of nucleic acid binding proteins. (TIF) [file pone.0180428.s006.tif]

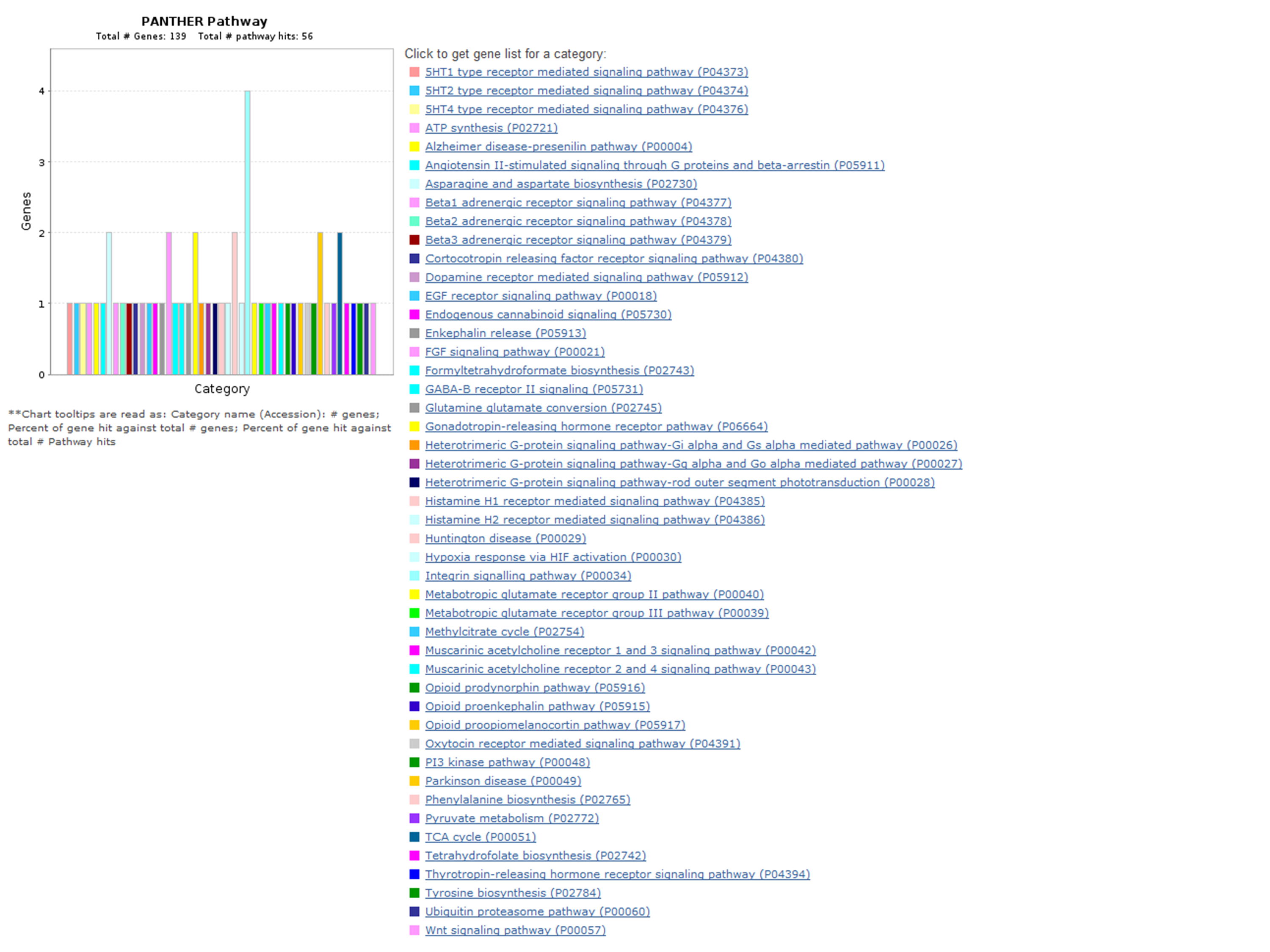

Supplement: S7 Fig — The most enriched pathway among the C1 cluster proteins was the integrin signaling pathway. (TIF) [file pone.0180428.s007.tif]

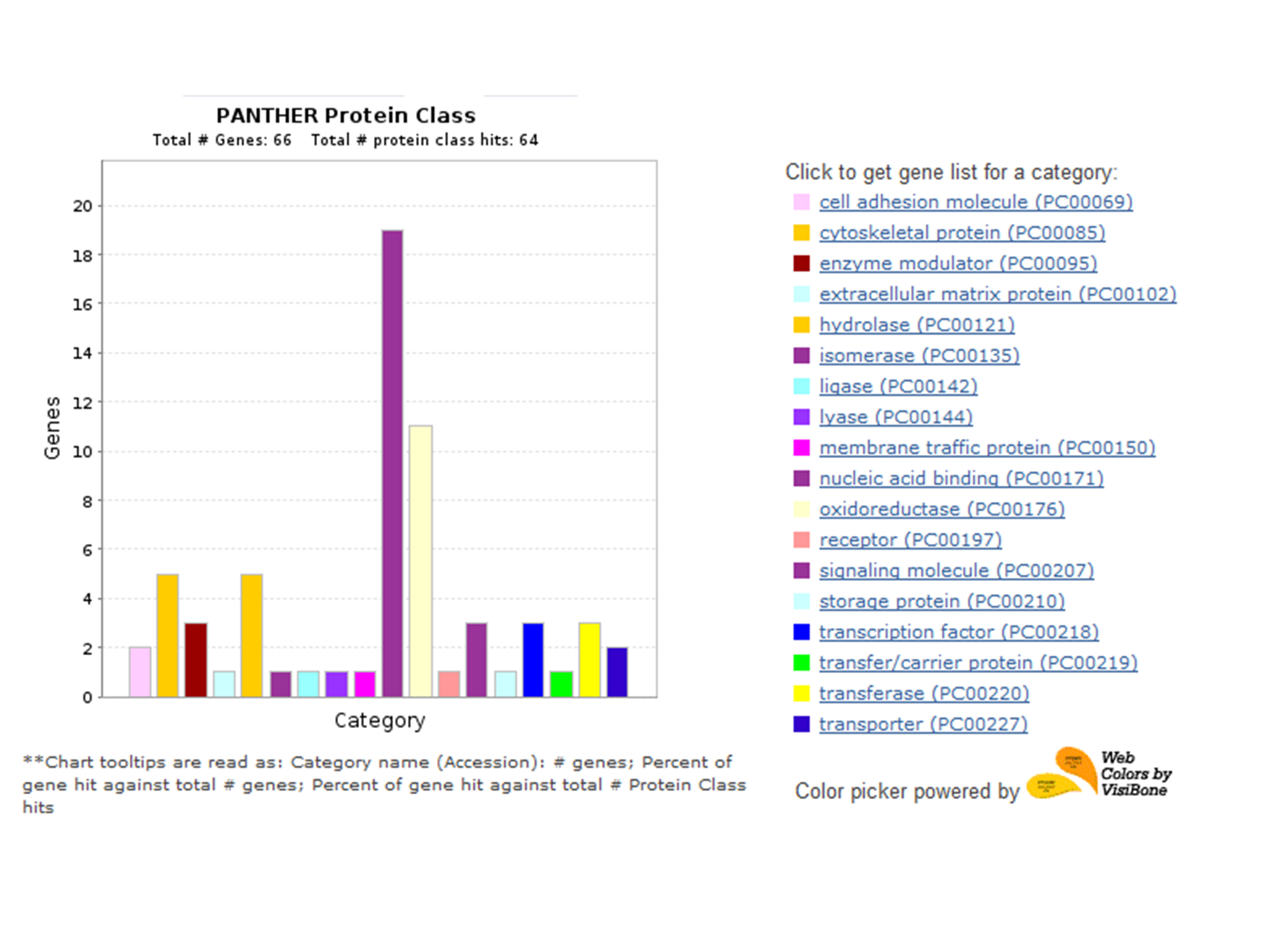

Supplement: S8 Fig — Most of the proteins from this cluster belong to the class of nucleic acid binding proteins. (TIF) [file pone.0180428.s008.tif]

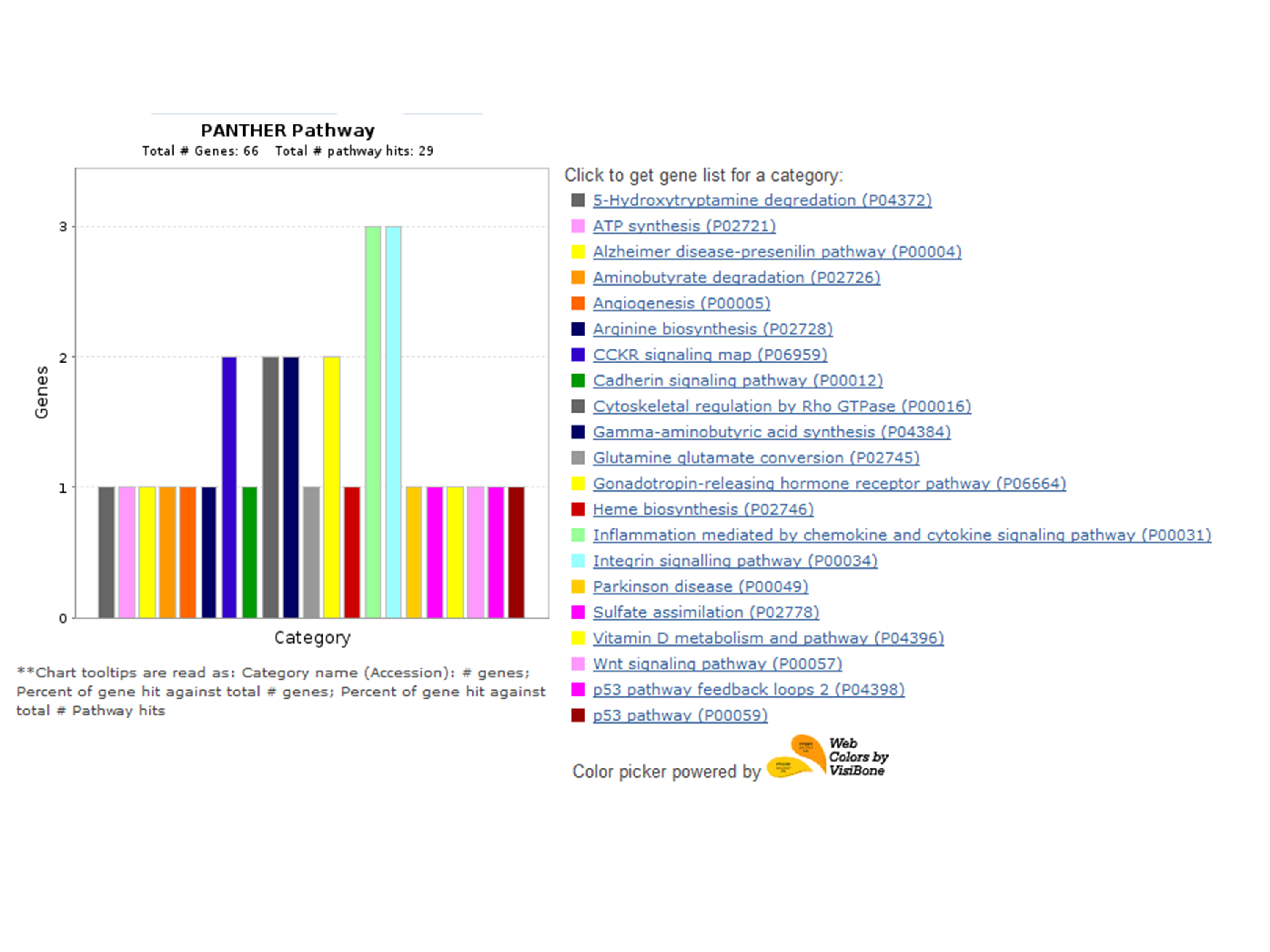

Supplement: S9 Fig — For the proteins of the cluster C2 two pathways were enriched: 1) inflammation mediated by the chemokines and cytokines signaling pathway, and 2) the integrin signaling pathway. (TIF) [file pone.0180428.s009.tif]

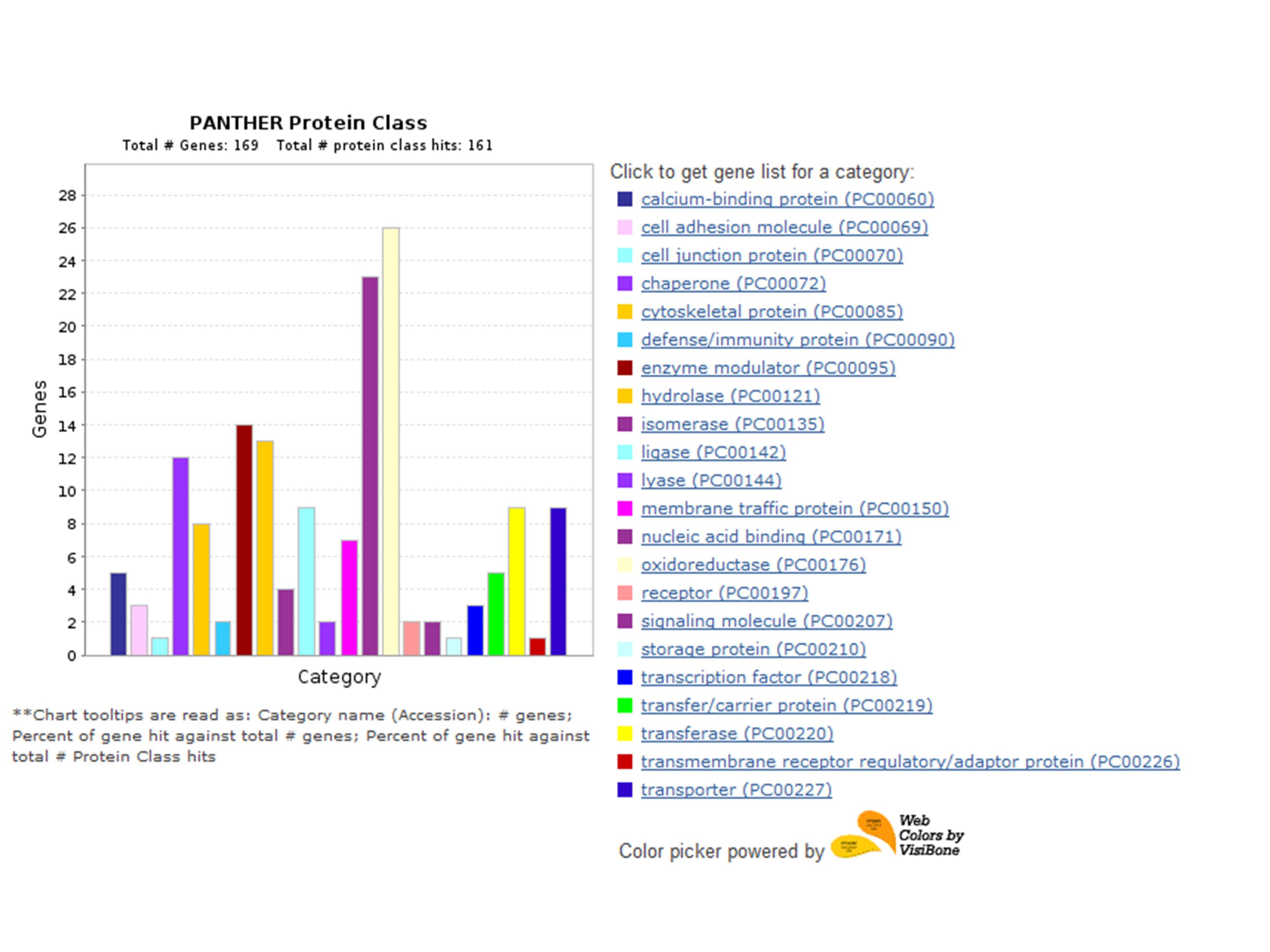

Supplement: S10 Fig — The most enriched for the proteins of this cluster was the oxidoreductase category. Also enriched were the nucleic acid binding and the enzyme modulator proteins. (TIF) [file pone.0180428.s010.tif]

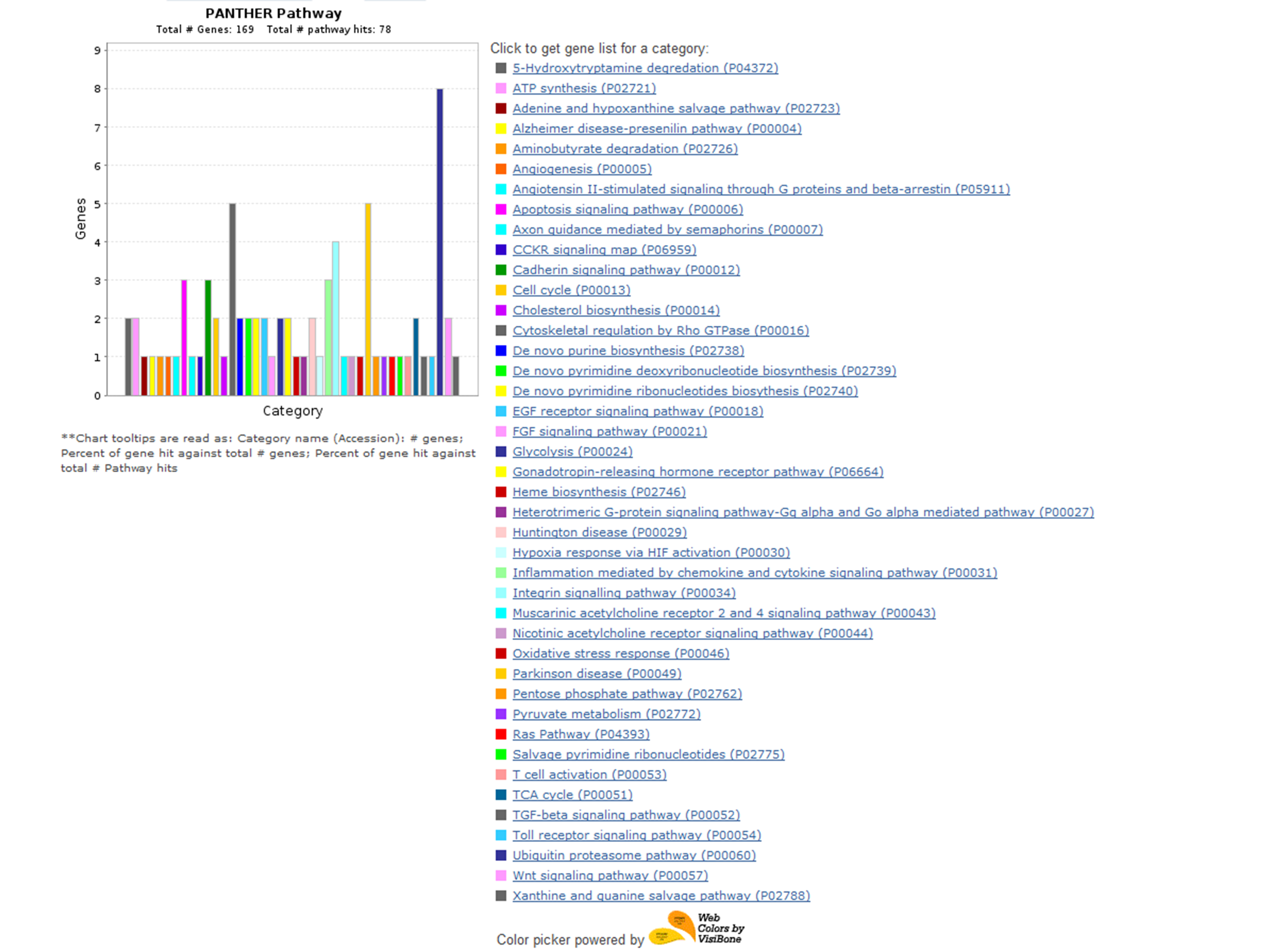

Supplement: S11 Fig — The short-living proteins belonged to the ubiquitin-proteasome pathway. (TIF) [file pone.0180428.s011.tif]
