# Supplementary material for: Predicting the protein half-life in tissue from its cellular properties
Source: PLoS One. 2017 Jul 18;12(7):e0180428. doi: 10.1371/journal.pone.0180428 (PMC5515413; doi:10.1371/journal.pone.0180428)
Supplement: S2 Table — (DOCX) [file pone.0180428.s013.docx]

S2 Table.

| Cluster |  | | | | | | |
| --- | --- | --- | --- | --- | --- | --- | --- |
|  | w^G^_Cell_half-life_ | w^G^_P_length_ | w^G^_P_abundance_ | w^G^_I_sequence_ | w^G^_mRNA_ | w^G^_Transcription_ | w^G^_Translation_ |
| C_1_ | 0.408 | 0.091 | 0.124 | 0.002 | 0.990 | 0.001 | 0.380 |
| C_2_ | 0.641 | 0.008 | 0.028 | 0.215 | 0.179 | 0.087 | 0.136 |
| C_3_ | 0.831 | 0.006 | 0.007 | 0.003 | 0.149 | 0.994 | 0.180 |
