# Supplementary material for: Predicting the protein half-life in tissue from its cellular properties
Source: PLoS One. 2017 Jul 18;12(7):e0180428. doi: 10.1371/journal.pone.0180428 (PMC5515413; doi:10.1371/journal.pone.0180428)
Supplement: S3 Table — Negatively-correlated protein properties (S1 Table) have 0 weights (e.g. wGmRNA in C1,C2,C3.). (DOCX) [file pone.0180428.s014.docx]

**S3 Table.**

| Cluster |  | | | | | | |
| --- | --- | --- | --- | --- | --- | --- | --- |
|  | w^G^_Cell_half-life_ | w^G^_P_length_ | w^G^_P_abundance_ | w^G^_I_sequence_ | w^G^_mRNA_ | w^G^_Transcription_ | w^G^_Translation_ |
| C_1_ | 0.470 | 0.129 | 0.089 | 0.001 | 0.0 | 0.0 | 0.0 |
| C_2_ | 0.644 | 0.0 | 0.175 | 0.0 | 0.0 | 0.533 | 0.0 |
| C_3_ | 0.852 | 0.0 | 0.001 | 0.0 | 0.0 | 0.0 | 0.295 |
