# Supplementary material for: Predicting the protein half-life in tissue from its cellular properties
Source: PLoS One. 2017 Jul 18;12(7):e0180428. doi: 10.1371/journal.pone.0180428 (PMC5515413; doi:10.1371/journal.pone.0180428)
Supplement: S4 Table — (DOCX) [file pone.0180428.s015.docx]

**S4 Table.**

| **Clusters** | **Common characteristics** |
| --- | --- |
| **C_1_** | 1. Largest intercept, largest and scattered protein half-lives.   b) Weakest correlation coefficient between tissue half-life and cell half-life.  c) Highest number of positively correlated protein properties (e.g. proteins from liver and heart). |
| **C_2_** | 1. Completely linear relationship between tissue half-life and cell half-life. 2. Strongest correlation coefficient between tissue half-life and cell half-life. |
| **C_3_** | 1. Smallest intercept and shortest protein half-lives. 2. Intrinsically disordered sequences and ubiquitinated sites have direct influence on the tissue half-life (e.g. proteins from liver and heart in Tables S1 and S11 respectively). |
