# Supplementary material for: Predicting the protein half-life in tissue from its cellular properties
Source: PLoS One. 2017 Jul 18;12(7):e0180428. doi: 10.1371/journal.pone.0180428 (PMC5515413; doi:10.1371/journal.pone.0180428)
Supplement: S5 Table — (DOCX) [file pone.0180428.s016.docx]

S5 Table.

| Cluster | w_c_ = Intercept (h) | Regression coefficient | P-value |
| --- | --- | --- | --- |
| C_1_ | 725.7196 | 0.1193 | 0.596 |
| C_2_ | 439.01688 | 0.28151 | 2.039e-08 |
| C_3_ | 330.8414 | 0.1830 | 0.00823 |
