# Supplementary material for: Predicting the protein half-life in tissue from its cellular properties
Source: PLoS One. 2017 Jul 18;12(7):e0180428. doi: 10.1371/journal.pone.0180428 (PMC5515413; doi:10.1371/journal.pone.0180428)
Supplement: S6 Table — (DOCX) [file pone.0180428.s017.docx]

S6 Table.

| Cluster | Cor(Tissue half-life (Y_c_), Cell half-life (X_c_)) | Cor(Tissue half-life (Y_c_), Protein length (PL)) | Cor(Tissue half-life (Y_c_), Protein abundance (PA)) | Cor(Tissue half-life (Y_c_), Intrinsically disordered sequence (ID)) | Cor(Tissue half-life (Y_c_), mRNA level (MR)) | Cor(Tissue half-life (Y_c_), Transcription rate (TR)) | Cor(Tissue half-life (Y_c_), Translation rate (TL)) |
| --- | --- | --- | --- | --- | --- | --- | --- |
| C_1_ | 0.06 | -0.05 | -0.09 | 0.02 | 0.007 | -0.02 | -0.05 |
| C_2_ | 0.59 | -0.08 | 0.21 | -0.22 | 0.02 | 0.0004 | 0.02 |
| C_3_ | 0.19 | 0.09 | 0.09 | 0.05 | 0.09 | -0.05 | 0.09 |
