# Supplementary material for: Predicting the protein half-life in tissue from its cellular properties
Source: PLoS One. 2017 Jul 18;12(7):e0180428. doi: 10.1371/journal.pone.0180428 (PMC5515413; doi:10.1371/journal.pone.0180428)
Supplement: S9 Table — C2 provides the best result. It has predicted 76% of protein half-lives within 10% deviation from the experimental value. (DOCX) [file pone.0180428.s020.docx]

S9 Table.

| Cluster | PCH | | | | | ACH | | | | | | | |
| --- | --- | --- | --- | --- | --- | --- | --- | --- | --- | --- | --- | --- | --- |
|  | Deviation | 5% | 10% | 20% | 30% | Deviation | 5% | 10% | | 20% | | 30% | |
| C_1_ | PE% | 5.2% | 18.9% | 43% | 79% | PE% | 5% | | 19% | | 43% | | 79% |
| **C_2_** | **PE%** | **41%** | **76%** | **100%** | **100%** | **PE%** | **41%** | | **76%** | | **100%** | | **100%** |
| C_3_ | PE% | 19% | 47% | 80% | 88% | PE% | 19% | | 47% | | 80% | | 88% |
