# Supplementary material for: Predicting the protein half-life in tissue from its cellular properties
Source: PLoS One. 2017 Jul 18;12(7):e0180428. doi: 10.1371/journal.pone.0180428 (PMC5515413; doi:10.1371/journal.pone.0180428)
Supplement: S10 Table — (DOCX) [file pone.0180428.s021.docx]

S10 Table.

| Cluster | w_c_ = Intercept (h) | Regression coefficient | P-value |
| --- | --- | --- | --- |
| C_1_ | 362.1962 | 0.6870 | 0.0004363 |
| C_2_ | 130.64420 | 1.13684 | 2.2e-16 |
| C_3_ | 52.54941 | 1.05460 | 2.2e-16 |
