# Supplementary material for: Predicting the protein half-life in tissue from its cellular properties
Source: PLoS One. 2017 Jul 18;12(7):e0180428. doi: 10.1371/journal.pone.0180428 (PMC5515413; doi:10.1371/journal.pone.0180428)
Supplement: S11 Table — (DOCX) [file pone.0180428.s022.docx]

S11 Table.

| Cluster | Cor(Tissue half-life (Y_c_), Cell half-life (X_c_)) | Cor(Tissue half-life (Y_c_), Protein length (PL)) | Cor(Tissue half-life (Y_c_) , Protein abundance (PA)) | Cor(Tissue half-life (Y_c_), Intrinsically disordered sequence (ID)) | Cor(Tissue half-life (Y_c_), mRNA level (MR)) | Cor(Tissue half-life (Y_c_), Transcription rate (TR)) | Cor(Tissue half-life (Y_c_), Translation rate (TL)) |
| --- | --- | --- | --- | --- | --- | --- | --- |
| C_1_ | 0.23 | 0.04 | 0.13 | 0.04 | 0.01 | 0.04 | -0.12 |
| C_2_ | 0.99 | 0.01 | 0.38 | 0.14 | 0.24 | -0.01 | -0.05 |
| C_3_ | 0.88 | -0.10 | 0.16 | -0.23 | -0.23 | -0.12 | -0.04 |
