# Supplementary material for: Predicting the protein half-life in tissue from its cellular properties
Source: PLoS One. 2017 Jul 18;12(7):e0180428. doi: 10.1371/journal.pone.0180428 (PMC5515413; doi:10.1371/journal.pone.0180428)
Supplement: S13 Table — Negatively-correlated protein half-life properties (e.g. S11 Table) have 0 weights. (DOCX) [file pone.0180428.s024.docx]

**S13 Table.**

| Cluster |  | | | | | | |
| --- | --- | --- | --- | --- | --- | --- | --- |
|  | w^G^_Cell_half-life_ | w^G^_P_length_ | w^G^_P_abundance_ | w^G^_I_sequence_ | w^G^_mRNA_ | w^G^_Transcription_ | w^G^_Translation_ |
| C_1_ | 0.091 | 0.001 | 0.002 | 0.0004 | 0.004 | 0.001 | 0.0 |
| C_2_ | 0.999 | 0.002 | 0.794 | 0.001 | 0.999 | 0.0 | 0.0 |
| C_3_ | 0.873 | 0.0 | 0.003 | 0.0 | 0.0 | 0.0 | 0.0 |
