# Supplementary material for: Predicting the protein half-life in tissue from its cellular properties
Source: PLoS One. 2017 Jul 18;12(7):e0180428. doi: 10.1371/journal.pone.0180428 (PMC5515413; doi:10.1371/journal.pone.0180428)
Supplement: S14 Table — C2 provides the best result. It has predicted 33% of protein half-lives within 10% deviation from the experimental value. (DOCX) [file pone.0180428.s025.docx]

S14 Table.

| Cluster | PCH | | | | | ACH | | | | | | | |
| --- | --- | --- | --- | --- | --- | --- | --- | --- | --- | --- | --- | --- | --- |
|  | Deviation | 5% | 10% | 20% | 30% | Deviation | 5% | 10% | | 20% | | 30% | |
| C_1_ | PE% | 7% | 12.5% | 30% | 54% | PE% | 7% | | 12.5% | | 30% | | 54% |
| **C_2_** | **PE%** | **19%** | **33%** | **59%** | **74%** | **PE%** | **19%** | | **33%** | | **59%** | | **74%** |
| C_3_ | PE% | 6% | 11.4% | 22% | 31% | PE% | 6% | | 11.4% | | 22% | | 31% |
