# Supplementary material for: Predicting the protein half-life in tissue from its cellular properties
Source: PLoS One. 2017 Jul 18;12(7):e0180428. doi: 10.1371/journal.pone.0180428 (PMC5515413; doi:10.1371/journal.pone.0180428)
Supplement: S15 Table — (DOCX) [file pone.0180428.s026.docx]

S15 Table.

| Protein Data Sets | Liver | | Brain | | Heart | |
| --- | --- | --- | --- | --- | --- | --- |
|  | cor(Tissue half-life, Ubiquitination) | cor(Cell half-life, Ubiquitination) | cor(Tissue half-life, Ubiquitination) | cor(Cell half-life, Ubiquitination) | cor(Tissue half-life, Ubiquitination) | cor(Cell half-life, Ubiquitination) |
| C_1_ | 0.008 | 0.026 | -0.033 | 0.196 | 0.153 | 0.035 |
| C_2_ | -0.159 | -0.133 | 0.040 | -0.111 | 0.244 | 0.270 |
| C_3_ | -0.116 | -0.084 | -0.0294 | 0.059 | -0.092 | -0.052 |
